# Lack of spontaneous age-related brain pathology in *Octodon degus*: a reappraisal of the model

**DOI:** 10.1038/srep45831

**Published:** 2017-04-04

**Authors:** Mathieu Bourdenx, Sandra Dovero, Marie-Laure Thiolat, Erwan Bezard, Benjamin Dehay

**Affiliations:** 1 Univ. de Bordeaux, Institut des Maladies Neurodégénératives, UMR 5293, F-33000 Bordeaux, France; 2 CNRS, Institut des Maladies Neurodégénératives, UMR 5293, F-33000 Bordeaux, France

## Abstract

Neurodegenerative diseases are characterized by the degeneration of specific brain areas associated with accumulation of disease-related protein in extra- or intra-cellular deposits. Their preclinical investigations are mostly based on genetically-engineered animals. Despite their interest, these models are often based on high level of disease-related protein expression, thus questioning their relevance to human pathology and calling for the alternate use of ecological models. In the past few years, *Octodon degus* has emerged as a promising animal model displaying age-dependent Alzheimer’s disease-related pathology. As neurodegenerative-related proteins often co-deposit in the brain of patients, we assessed the occurrence of α-synuclein-related pathology in this model using state-of-the-art immunohistochemistry and biochemistry. Despite our efforts and in contrast with previously published results, our study argues against the use of *Octodon degus* as a suitable natural model of neurodegenerative disorder as we failed to identify either Parkinson’s disease- or Alzheimer’s disease-related brain pathologies.

Neurodegenerative diseases are characterized by the presence of intracellular or extracellular proteinaceous aggregates such as senile plaques in Alzheimer’s disease (AD) and Lewy bodies (LBs) in Parkinson’s disease (PD)^1^. LBs are notably composed of α-synuclein (α-syn), a presynaptic protein of which mutations induce hereditary parkinsonism^1^,^2^. Besides protein aggregation, PD is characterized by the loss of several neuronal populations including dopaminergic neurons in the *substantia nigra* (SN) and dopaminergic fibers in the striatum. This neuronal cell death induces a dramatic loss of dopamine in the striatum, which is responsible for most of the motor symptoms. In the past two decades, α-syn has been increasingly linked to PD-related neurodegenerative mechanisms, including cell-autonomous and non-cell autonomous dysfunctions, making it a major target in the treatment of PD^1^,^2^.

Our ability to identify and test new therapeutic agents is directly based on the validity of our preclinical models. In the past 50 years, numerous models for PD have been characterized ranging from acute dopaminergic depletion to transgenic mice and viral-mediated overexpression in mammals^3^,^4^. α-Syn-based models, especially those relying on intracerebral delivery of overexpressing viral vectors, produce interesting phenotypes, by combining nigrostriatal degeneration and some protein aggregation, but are still unable to recapitulate the complexity of the human pathology. Naturally-occurring or ecological models of PD recapitulating both a motor phenotype associated with dopaminergic neurodegeneration and α-syn aggregation would be a substantial addition to research in the field.

Over the past decade, several contributions have described that *Octodon degus*, a South American rodent, would display amyloid-β and tau deposits as well as post-synaptic dysfunction^5^,^6^,^7^. This rodent presents an interesting homology with human amyloid-β (Aβ) sequence differing in only one amino-acid^8^, a feature similarly displayed by α-syn with a 96% predicted sequence homology (96%) compared to human sequence (Supp. Table 1). In the brain of patients suffering from various neurodegenerative disorders, accumulation of neurodegenerative disease-related proteins such as Aβ, tau or α-syn often co-occur in the different diseases^9^. For instance, while Aβ-related pathology is often found in most elderly’s brain, the tiny ratio between tau and α-syn seems to determine the final diagnosis between pure AD and pure dementia with LB^10^. Because *Octodon degus* thus appeared as a putative candidate for being an ecological model of synucleinopathies, we here assessed age-associated α-syn pathology in the brains of young and aged octodons.

## Results

### Absence of PD-related brain pathology

**Figure 1:**
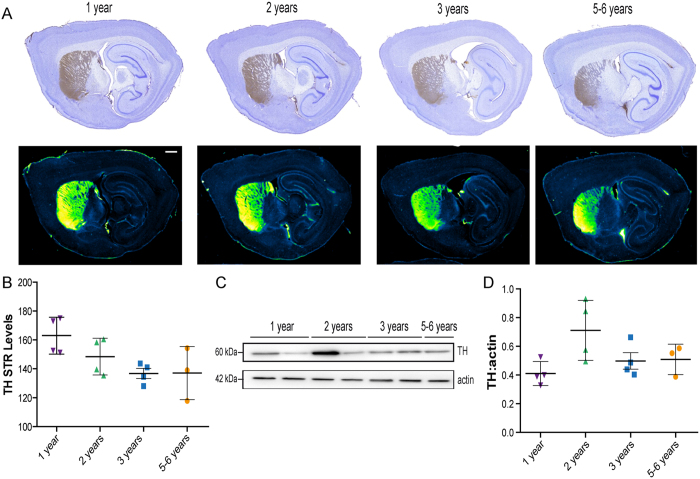
Absence of age-dependent nigrostriatal pathway neurodegeneration in wild-type *Octodon degus*. (**A**) Representative photomicrographs of striatal tyrosine hydroxylase (TH) immunostaining in sagittal section of octodons. In the lower panel, Green fire blue LUT (lookup table) was applied to scanned section to enhance contrast and highlight the absence of difference between groups. (**B**) No significant decrease of mean grey values of striatal TH immunoreactivity [F_(3,11)_ = 3.561, *p* = 0.0509]. (**C**,**D**) TH immunoblot levels in midbrain protein homogenates from octodons at different ages. Scale bar: 1 mm.

We first assessed the integrity of the nigrostriatal pathway that degenerates in PD^2^. To do so, we investigated tyrosine hydroxylase (TH) immunoreactivity levels in the striatum (Fig. 1A,B) that did not show an age-dependent reduction of signal. Then, the expression of TH in the midbrain was studied and revealed the same lack of decrease over-time (Fig. 1C,D).

**Figure 2:**
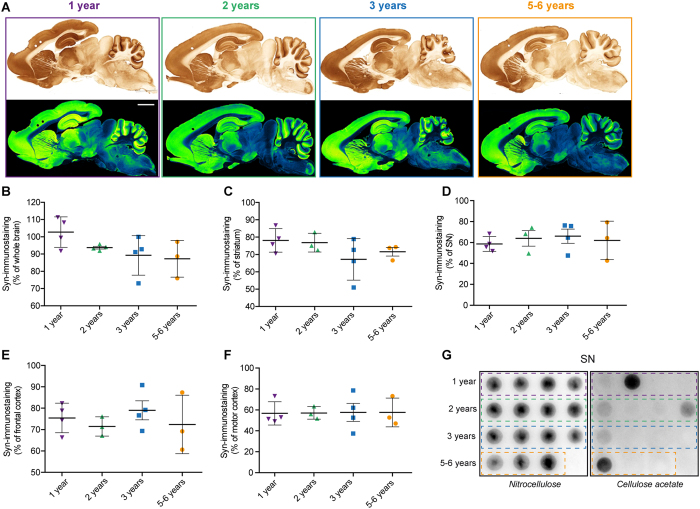
Distribution of α-synuclein in wild-type *Octodon degus* brains. (**A**) Sagittal brain section of octodons at different ages showing distribution of α-synuclein (α-syn) immunoreactive PD-related regions. In the lower panel, Green fire blue LUT (lookup table) was applied to scanned section to enhance contrast and highlight the absence of difference between groups. (**B**–**F**) Surface quantification of the α-syn immunostaining in whole brain (**B**), at striatal (**C**) and mesencephalic (**D**) levels as well as in the frontal (**E**) and motor cortex (**F**) of octodons. (**G**) Midbrain protein extracts were analyzed by filter retardation assay either on nitrocellulose membrane (left) or cellulose acetate membrane (right) and revealed by immunoblot with α-syn antibody. Scale bar: 3 mm.

**Figure 3:**
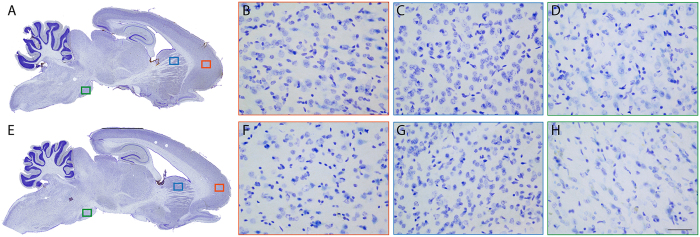
Absence of aggregated α-synuclein in *Octodon degus* brains. (**A**,**E**) Sagittal brain sections of young (**A**) and aged (**E**) octodons showing the distribution of proteinase-K (PK) resistant α-synuclein (α-syn) immunostaining. Representative pictures of PK-resistant α-syn staining in 3 brain regions: cortex (**B**,**F**), dorsal striatum (**C**,**G**), and *substantia nigra* (**D**,**H**). Scale bar: 100 μm.

We next focused on regional α-syn expression pattern that is known to increase in brains from PD patients^1^,^2^. Whole brain and four PD-related brain regions were investigated: striatum, SN, frontal and motor cortex (Fig. 2A–F). Regional α-syn immunostaining quantification did not allow to identify a local accumulation of α-syn over time. To get insight on the ratio of soluble versus aggregated α-syn, we used a filter retardation assay^11^. This assay is based on the finding that the SDS-insoluble protein aggregates obtained are retained on a cellulose acetate filter, whereas the soluble are not^11^. Thus, we performed this assay twice, first on a nitrocellulose membrane (Fig. 2G left panel) and then on a cellulose acetate membrane (Fig. 2G right panel) specifically retaining protein aggregates. In this quantitative assay, we detected on mesencephalic total protein extracts the presence of high molecular weight insoluble α-syn aggregates^11^,^12^, but no specific age-related pattern could be identified (Fig. 2G). Then, tissues sections were subject to Proteinase-K (PK) digestion and to immunohistochemistry with α-syn antibody (Fig. 3). In the same previously examined three regions, no PK-resistant α-syn-positive immunostaining could be identified suggesting the absence of α-syn β-sheet-rich aggregates.

**Figure 4:**
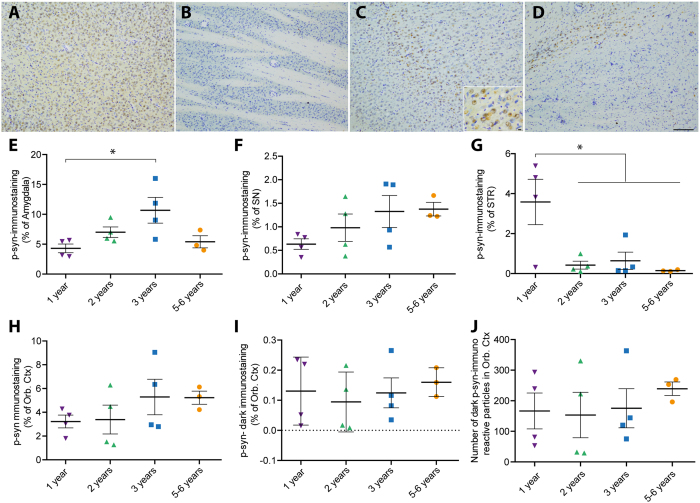
Distribution of phosphorylated α-synuclein in *Octodon degus* brains. (**A**–**D**) Representative images of phosphorylated α-synuclein (p-syn) in different brain regions of 3 years-old octodons (**A**) Amygdala; (**B**) Orbito-frontal cortex (Orb Ctx); (**C**) Striatum (STR); (**D**) *substantia nigra* (SN). Inset is a high magnification picture in striatum. (**E**–**J**) Bottom panels display surface quantification of the p-syn immunostaining in amygdala (**E**), SN (**F**), STR (**G**), Orb Ctx (**H**–**J**). Significant age effect was observed in amygdala [F_(3,11)_ = 4.275, *p* = 0.0314] and STR [F_(3,11)_ = 5.977, *p* = 0.0114]. (**J**) Number of dark spot of p-syn immunostaining in the orbital cortex. Scale bar: 100 μm, high magnification: 10 μm. *p < 0.05.

The most common age-related post-translational modification of α-syn is serine-129 phosphorylation^13^. Phosphorylated α-syn (p-syn) is widely considered as a pathological status marker^1^,^2^. We therefore investigated the putative accumulation of p-syn in four brain regions of interest: amygdala, orbito-frontal cortex, striatum and SN (Fig. 4). Although no clear stereotypical age-related brain pattern was observed across all age groups, we found two brain regions that showed significant associations with age, in the amygdala (Fig. 4E) and in the striatum (Fig. 4G). Surprisingly, at high magnification, dark p-syn dots could be observed in the orbito-frontal cortex region (Fig. 4C). Specific quantification of this darker signal did not show any age-dependent accumulation (Fig. 4I,J).

Altogether, these data show neither overt age-related lesion of the nigrostriatal pathway nor age-related α-syn pathology.

### Accumulation of ubiquitinated proteins in the SN of aged octodons

**Figure 5:**
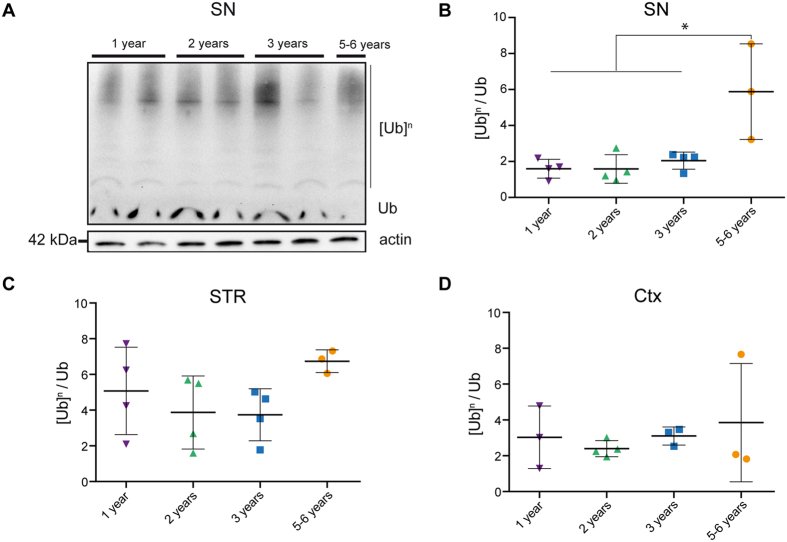
Accumulation of ubiquitinated proteins in aged octodons. (**A**) Representative ubiquitin immunoblot in the mesencephalon. [Ub]^n^: poly-ubiquitinated proteins. Ub: monomeric ubiquitin. (**B**–**D**) Levels of poly-ubiquitinated proteins in tissue homogenates from (**B**) the *substantia nigra* (SN), (**C**) the striatum (STR), (**D**) cortex (Ctx). SN present a significant age-dependent accumulation of poly-ubiquitinated proteins [F_(3,11)_ = 8.703, *p* = 0.003]. *p < 0.05.

Most neurodegenerative disorders are associated with the accumulation of ubiquitin-positive protein aggregates. We therefore investigated the occurrence of age-related accumulation of poly-ubiquitinated proteins in three regions of the brain of octodons: SN, striatum and frontal cortex (Fig. 5). We found a modest age-related accumulation of poly-ubiquitinated proteins only in the SN of aged (5–6 years-old, p < 0.05) octodons (Fig. 5A,B) but neither in the striatum nor in the cortex (Fig. 5C,D).

### Absence of AD-related brain pathology

**Figure 6:**
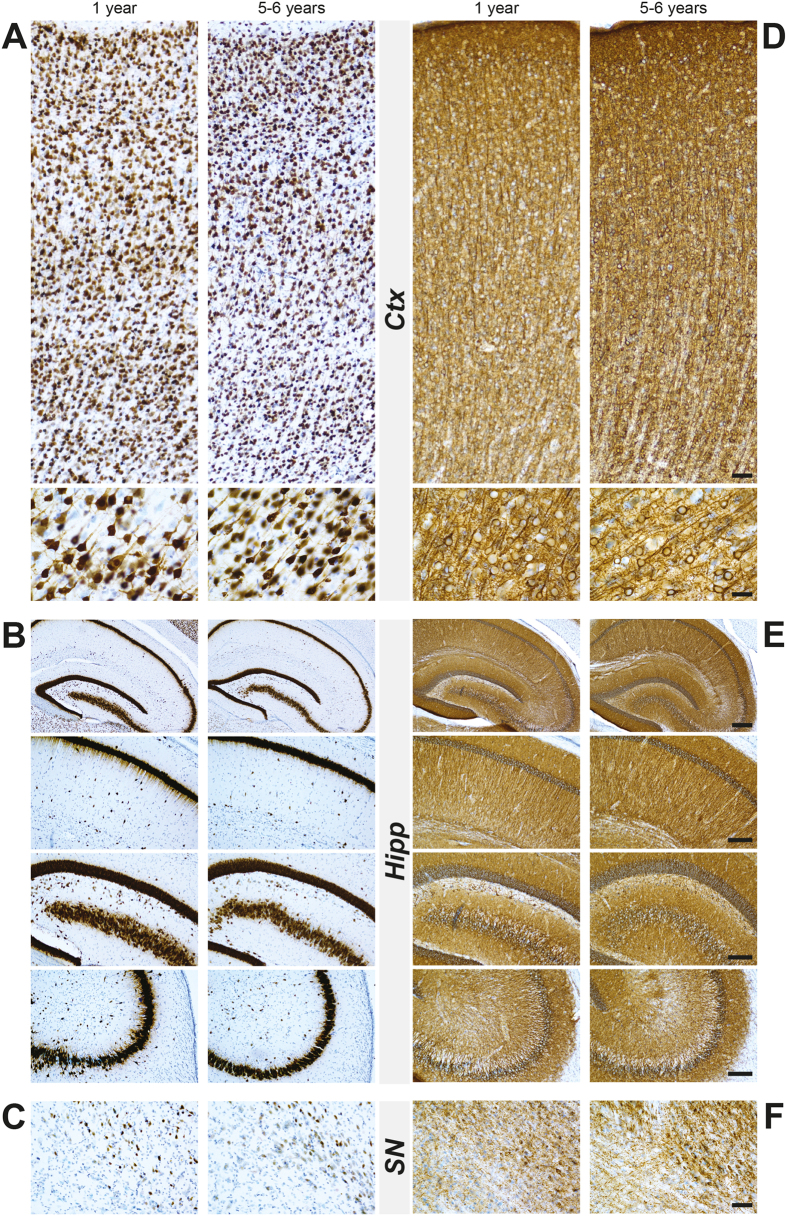
Profile of NeuN and Map2 immunostaining in the cortex, hippocampus and substantia nigra in wild-type young (1-year-old) and aged (5–6 years old) octodons. (**A**–**C**) Show representative cortical (**A**), hippocampal (**B**), nigral (**C**) images with low and high magnifications in young and aged octodons without clear changes in average density of neurons. (**D**–**F**) Show representative images of Map2 immunostaining in cortical (**D**), hippocampal (**E**), and nigral (**F**) areas from young and aged octodons. Scale bar (from the top to the bottom): (**A**–**D**) 40 μm; 20 μm; (**B**–**E**) 200 μm; 100 μm; 100 μm; 100 μm; (**C**–**F**) 40 μm.

**Figure 7:**
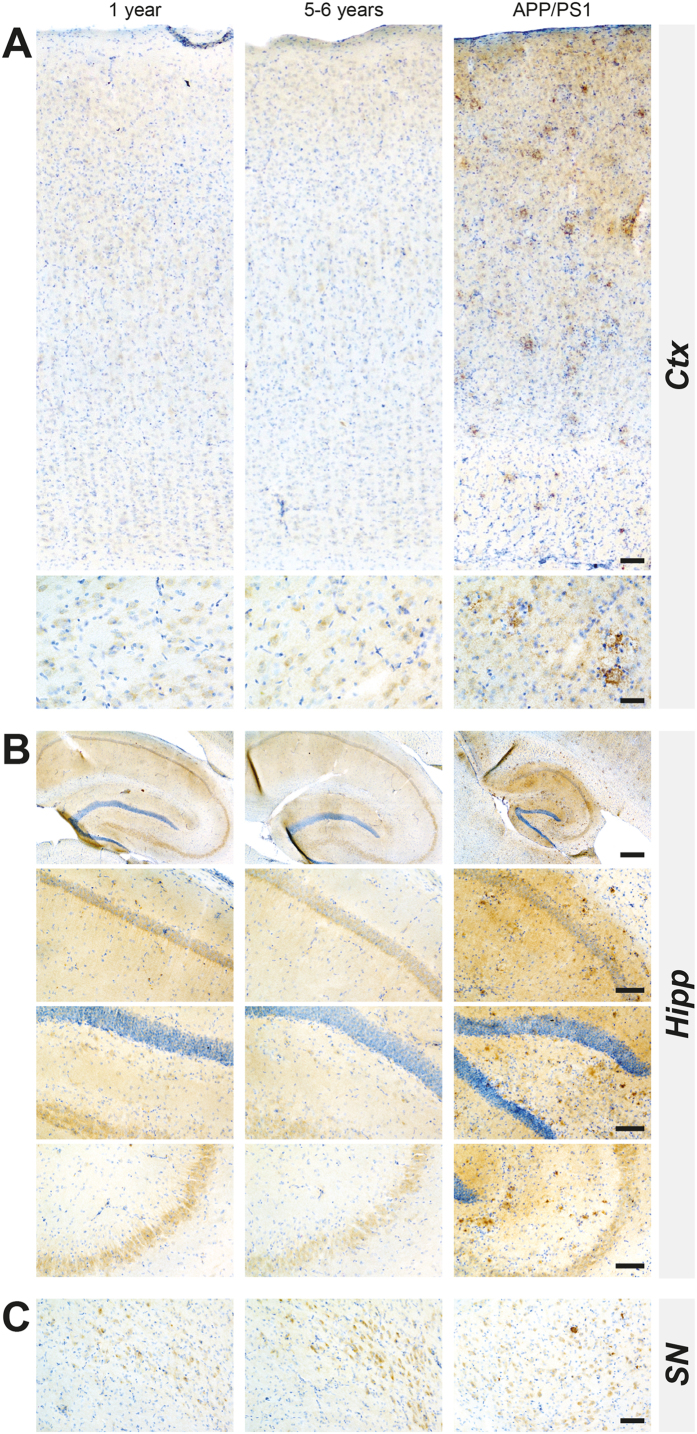
Comparison of β-amyloid pathology in the cortex, hippocampus and substantia nigra in wild-type young (1-year-old), aged (5–6 years old) octodons and in APP/PS1 transgenic mice (9 months). (**A**) Cortex (Ctx); (**B**) Hippocampus (Hipp); (**C**) substantia nigra (SN). Scale bar (from the top to the bottom): (**A**) 40 μm; 20 μm; (**B**) 200 μm; 100 μm; 100 μm; 100 μm; (**C**) 40 μm.

Our investigation was prompted by previous reports suggesting that *Octodon degus* might be a spontaneously occurring model of AD^5^,^6^,^7^,^14^. Since we did not detect overt synucleinopathy or nigrostriatal lesion, we also characterized the AD-related pathology, in an attempt to replicate previous findings. We focused on two brains regions commonly affected in AD: hippocampus and cortex, as reported affected in previous reports^15^. We first examined the occurrence of cortical, hippocampal or nigral neurodegeneration by immunohistochemistry with pan-neuronal markers, neuronal nuclear antigen (NeuN) and microtubule-associated protein-2 (Map2) (Fig. 6). Aged animals displayed a small decrease in NeuN staining in both cortical and hippocampal region without a specific pattern (Fig. 6A–C). High magnification investigations revealed a normal neuronal morphology (Fig. 6A–C). We did not observe any age-related abnormal patterns of Map-2 immunolabeling in cortical, hippocampal and nigral regions (Fig. 6D–F). We then examined the amyloid pathology by focusing on the presence of Aβ oligomers by ELISA (Supp. Fig. 1). We failed to identify age-dependent variation of Aβ40 in cortical layers (Supp. Fig. 1A) and hippocampus (Supp. Fig. 1B). Finally, we performed a sensitive immunohistochemistry to detect Aβ deposition^16^ (Fig. 7) and/or hyperphosphorylated tau staining (Supp. Fig. 2). Direct comparison of young (1year-old) and aged (5–6 years-old) octodons (Fig. 7) strikingly shows a lack of extracellular deposit such as Aβ plaques in cortical and hippocampal regions, compared to APP/PS1 mice, a classic transgenic mouse model of AD (Fig. 7). Similarly, no intracellular deposits such as neurofibrillary tangles and hyperphosphorylation of tau could be observed in young or aged animals (Supp. Fig. 2).

### Multivariate analysis of age-dependent pathology

**Figure 8:**
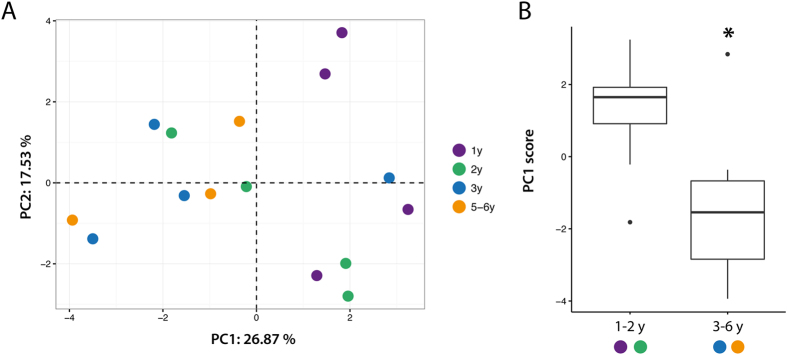
Multivariate analysis highlights aged-dependent pathology. (**A**) Principal component (PC) analysis was applied on 18 parameters measured for each animal (n = 15). Each animal is represented as a colored dot in the new space created by PC1–2. (**B**) Boxplot represent mean PC1 score of 1–2 years-old compared to 3–6 years-old animals. *p < 0.05 Two sample t-test.

To further analyze age-dependent phenotypes, we used principal component (PC) analysis on all measured variables (total number of variables = 18 (Supp. Table 2)) (Fig. 8A). This method is used to create principal components that are linear combinations of measured variables to emphasize variations in the dataset and allow to unravel overall patterns. Careful examination of the location of each animal in the space create by PC1 and PC2 suggest that, as reported by Ardiles *et al*.^5^, we can consider two age-groups (1–2 and 3–6 years-old) (Fig. 8B). Interestingly, individual scores along PC1 (according for 26.87% of the overall variance) showed a significant difference between “young” and “old” groups (t_13_ = −2.6162, *p* = 0.021) (Fig. 8A). The three strongest contributors of PC1 were the overall α-syn brain levels (Fig. 2B), α-syn level in the striatum (Fig. 2C) and p-syn in the SN (Fig. 4F). This analysis suggest that an age-related phenomenon can be unravel through multivariate analysis.

Altogether these data suggest that no AD-related pathology can be found in aged octodons, in conflict with previous reports^5^,^6^,^7^, but in accordance with a more recent contribution^8^.

## Discussion

We here assessed the relevance of *Octodon degus* as a suitable natural model of neurodegenerative disorders associated with protein inclusion formation. We considered four groups of age (i.e. 1, 2, 3, 5–6 years-old) and systematically assessed the occurrence of nigrostriatal degeneration, α-syn phosphorylation and aggregation, accumulation of poly-ubiquitinated proteins and occurrence of Aβ- and tau-related pathologies. Overall, our investigation has not unveiled any specific pattern of age-related pathology therefore questioning the relevance of such rodent as a natural model for age-related disorders. Interestingly, a recent study came to the same conclusions with the same groups of age while focusing on AD-related pathology^8^. Although the common max life expectancy is 3–4 years in the wild, captivity allows prolonged life span up to 5–8 years, thus suggesting that our age-groups are relevant for the study of age-related phenomenons^8^,^17^.

Neurodegenerative disorders are and will increasingly be a significant burden for the society as these age-related disorders require intensive care^1^. Thus, the need for relevant disease models to test putative therapeutic strategies is strong. In the field of PD, we can roughly consider three types of models: toxin-based models, transgenic models (either classical strategy or viral-vector mediated overexpression of disease relevant protein) and the most recent ones are based on injection of misfolded α-syn^3^,^4^. High levels of overexpression are often needed to obtain significant levels of neurodegeneration and protein aggregation thus questioning the relevance of such models. Such high overexpression levels are often associated with a downregulation of the endogenous protein^18^. Recent models based on the injection of misfolded aggregates are promising but their predictive value remain to be established. Natural or ecological models are therefore attractive alternatives. As an example, several groups, including us, have reported spontaneous, although modest, age-related brain pathology (accumulation of Aβ-positive deposits, tau-related pathology and α-syn-related pathology) in *Microcebus murinus* (mouse lemur primate)^19^,^20^,^21^.

Several papers have documented age-related dysfunctions and brain pathologies in aged octodons^5^,^6^,^7^. Behavioral deficits in cognitive tasks such as novel object recognition have been reported as well as post-synaptic dysfunction, accumulation of Aβ-positive and phosphorylated-tau-positive deposits^5^,^6^. More recently, astrocytic activation and oxidative stress have also been reported^7^. Interestingly, Ardiles and collaborators described that most age-related changes occurred between 12 and 36 months^5^. However, most of these deficits could not be identified in our cohort as well as in, a recently published independent study that also failed to identify AD-related brain pathology^8^.

However, to go beyond the gross changes, we decided to conduct multivariate analysis to unravel complex phenotypes thanks to linear combination of variables (Fig. 8). The three main contributors of PC1 nicely depict what we could expect from a PD model. Although some interesting phenotype arise from multivariate analysis, we failed to replicate most of the results of the previous anatomo-pathological studies. As noted by Steffen *et al*., a difference in the origin of the animals could explain such discrepancies^8^. Indeed, most published studies used a wide variety of source for the animals including animals that were caught from the wild^5^,^6^,^7^,^8^ while animals of the present study come from a breeding facility that allows precise date of birth as well as homogeneity in individual histories.

Ageing is a process that affects each individual but only some of us will develop age-related diseases such as neurodegenerative disorders. It is therefore difficult to decipher between normal ageing and pathological ageing. The most common characteristic among neurodegenerative disorders is the presence of protein aggregates^1^. However, 8 to 17% of people over 60 display LB pathology without another clinical phenotype^22^. Although such incidental brain pathology is often considered as a prodromal phase of neurodegenerative diseases, the fact that such individual will indeed convert to a disease state later-on remain without clear evidence. Ardiles *et al*. highlighted in their paper that “approximately 25% of individuals […] exhibit ‘impaired’ performance on [tests]”. This observation is coherent with what we could expect from a natural model of a given age-related pathology i.e. only a fraction of individual will express the pathology, as in Humans. However, the global trend that we observed (Fig. 8) and the very small variability displayed for all histology measurements reported in most of the papers about octodons suggest that we are facing a population-wide effect. This argues against octodons being a suitable natural model of AD or PD but it might be a highly valuable model for age-related processes.

In conclusion, we performed a thorough analysis of the occurrence of AD and PD brain pathology in young and aged octodons. Our study is entirely consistent with a recently published independent study^8^ and accordingly argues against the use of *Octodon degus* as a natural model of neurodegenerative disorders but supports its use as an animal model of ageing.

## Materials and Methods

### Animals

Octodons were housed under controlled conditions of humidity, temperature, and light (12-h light/12-h dark cycle, lights on at 8.00 am); food and water were available ad libitum. Experiments were carried out in accordance with European Communities Council Directive of 3 June 2010 (2010/6106/EU) on the protection of animals used for scientific purposes. The Institutional Animal Care and Use Committee of Bordeaux (CE50) approved experiments under the license number 5012099-A. Animals were purchased from the research animal facility of the University of Alicante (Alicante, Spain). *Octodon degus* at different ages have been used to reach a statistically significant group of animals of young (1 year old, n = 4), adult (2 years old, n = 4; 3 years old, n = 4), aged (5–6 years old, n = 3). Octodons were terminated with pentobarbital overdose (100 mg/kg i.p.) and intracardially perfused with 0.9% saline solution (containing 0.25% heparin). Brains were removed quickly after death. Each brain was then dissected along the midline. The right hemisphere was post-fixed for five days in 4% paraformaldehyde, cryoprotected in phosphate buffered saline (PBS) containing 20% sucrose before being frozen by immersion in a cold isopentane bath (−50 °C), and stored immediately at −80 °C until sectioning. Brains were sectioned in a Leica CM3050S cryostat (Leica Microsystem, Wetzlar, Germany) at −20 °C. Right hemisphere was cut on a cryostat into 50 μm-thick free-floating sagittal sections and stored in PBS containing 0.2% sodium azide at 4 °C until use. The left hemisphere was frozen freshly by immersion in cold isopentane bath at −50 °C during at least 5 min, and stored immediately at −80 °C for biochemistry investigation. To do so, left hemispheres were cut on a cryostat into 300 μm-thick sections and selected brain areas were punched. Tissues punches were then stored at −80 °C until further use.

### Histopathological analysis

#### Extent of lesion

To assess the integrity of the nigrostriatal pathway, tyrosine hydroxylase (TH) immunohistochemistry was performed on striatal free-floating sagittal sections. Briefly, sections were incubated with a mouse monoclonal antibody raised against human TH (Millipore, MAB318, 1:5000) performed as previously reported^23^. To characterize the pattern of dopaminergic cell loss in the SN, we processed mesencephalon extracts (20 μg) by western-blot assay under standard conditions and probed with the same TH antibody.

#### Investigation of brain pathology

For Neuronal Nuclear antigen (NeuN) and Microtubule-Associated Protein 2 (Map2) staining, sagittal free-floating sections of representative levels at different ages were incubated together with either NeuN (Millipore, MAB369, 1:500) or Map2 (Millipore, MAB3414, 1:500) mouse antibodies for one night at room temperature, washed in PBS and incubated with an anti-mouse peroxidase EnVision^TM^ system (DAKO, K400311) followed by DAB visualization. Sagittal sections were mounted on gelatinized slides, counterstained with 0.1% cresyl violet solution, dehydrated and coverslipped. Synucleinopathy has been assessed with rodent α-syn (BD Transduction, 610787, 1:1000) and p-syn (Wako, 015–2191, 1:5000) immunostaining as we previously reported^12^,^23^. Briefly, selected sections were specifically identified and incubated in a same well to allow direct comparison of immunostaining intensity. Sections were incubated over-night at room temperature with the aforementioned antibodies. The following day, revelation was performed with anti-specie peroxidase EnVision system (DAKO) followed by 3,3′-diaminobenzidine (DAB) incubation. Sections were then mounted on gelatinized slides, dehydrated, counter-stained if necessary and cover-slipped until further analysis. Grey level quantification or immunostaining-positive surface quantification were performed as previously described^23^. To assess the presence of amyloid **β** extracellular deposits, the mouse 6E10 anti **β**-amyloid clone antibody (Eurogentec, 6E10, 1:1000) was used as previously reported^16^. Briefly, 50 μm sagittal free-floating sections of representative levels of octodons at different ages and APP/PS1 at age 6–9 months were pretreated with a further triple combination of digestion with 1 μg/mL proteinase K solution (10 min RT), water-bath EDTA heating (20 min −80 °C) and 98% formic acid treatment (1 min RT). After each antigen retrieval treatment, sections were washed with tap water for at least 5 min and then incubated in distilled water for at least 5 min. The 6E10 antibody was incubated one night at RT and revealed by the anti-mouse peroxidase EnVision^TM^ system (DAKO, K400311) as described for NeuN and Map2 immunostainings. Hyper-phosphorylated tau (p-tau) deposits were stained with AT8 antibody (ThermoFisher, 1:1000).

#### Proteinase-K digestion assay

Fifty-micrometer thick sections were washed in PBS prior to incubation in Proteinase-K (PK, 1 μg/ml) at room temperature for 10 minutes. Sections were then washed 3 times in PBS and immunostained for α-syn as indicated in the previous Materials and Methods section.

### Biochemical analysis

#### Western blot analysis of ubiquitinated proteins

Immunoblot analyses were performed on mesencephalon, striatum and cortex. Tissue patches were homogenized in RIPA buffer (50 mM Tris pH 8.0, 150 mM NaCl, 1.0% Triton X-100, 0.5% sodium deoxycholate, 0.1% sodium dodecyl sulfate) with a protease inhibitor cocktail tablet (Complete Mini, Roche Diagnostics). Western Blots were run in all conditions from 20 μg of protein separated by SDS-PAGE. For detection of ubiquitinated proteins, proteins were transferred on polyvinylidene fluoride membranes (Millipore) and subjected to Western blot analysis using a rabbit anti-Ubiquitin 1:1000 (Sigma U5379). Signals were revealed with horseradish peroxydase-conjugated secondary antibodies. Anti-actin antibody (1:5000; Sigma) was used as loading control. Signals per lane were quantified using ImageJ and a ratio of signal on loading per animal was performed and used in statistical analyses.

#### Dot blot analysis of α-synuclein

This technique was performed as we previously described^11^,^12^. After heating at 100 °C for 5 min, 20 μg of protein extract was diluted in buffer (25 mM Tris-HCl, 200 mM Glycine, 1% SDS) and filtered through either a nitrocellulose membrane or an acetate cellulose membrane (Bio-Rad, 0.2 μm pore size). Membranes were then saturated in 5% dry-milk prior to incubation with α-syn (BD Transduction, 610787, 1:1000) antibody. Revelation was done as described in the previous Materials and Methods section.

#### Quantification of Aβ (1–40) peptide

Analyses were performed on hippocampus and cortex. Tissue patches were homogenized in lysis buffer containing 20 mM HEPES, 0.15 mM NaCl, 1% triton x100, 1% deoxycholic acid, 1% SDS, pH 7.5 and supplemented with a protease inhibitor cocktail tablet (Complete Mini, Roche Diagnostics). Protein amounts of hippocampal and cortical homogenates were determined by the bicinchoninic acid assay (#23227; Thermo Scientific, Waltham, MA) and quantification of Aβ (1–40) peptide was performed using the Human/Rat β-amyloid ELISA kit (Wako chemicals #292–62301) according to the manufacturer’s instructions. Aβ concentration in samples was determined by comparison to a standard curve (0–100 pmol/l). The absorbance at 450 nm was read using a microplate reader.

### Statistical analysis

All values are expressed as the mean ± standard error of the mean. For all experiments, comparisons among means were performed by using One-way analysis of variance (ANOVA) followed, if appropriate, by a pairwise comparison between means by Tukey *post-hoc* analysis. Principal component (PC) analysis was performed in R statistical environment (version 3.3.1)^24^ using FactoMineR package (version 1.33)^25^. Missing values (9 out of 270–3,33%) were imputed thanks to the K-Nearest Neighbour method. Comparison between PC scores for each age group was performed using Student *t* test. In all analyses, statistical significance was set at p < 0.05.

## Additional Information

**How to cite this article**: Bourdenx, M. *et al*. Lack of spontaneous age-related brain pathology in *Octodon degus*: a reappraisal of the model. *Sci. Rep.* **7**, 45831; doi: 10.1038/srep45831 (2017).

**Publisher's note:** Springer Nature remains neutral with regard to jurisdictional claims in published maps and institutional affiliations.

## Supplementary Material

Supplementary Information
