# Supplementary material for: Lack of spontaneous age-related brain pathology in Octodon degus: a reappraisal of the model
Source: Sci Rep. 2017 Apr 4;7:45831. doi: 10.1038/srep45831 (PMC5379186; doi:10.1038/srep45831)
Supplement: Supplementary Information [file srep45831-s1.pdf]

Lack of spontaneous age-related brain pathology in *Octodon degus*: a reappraisal of the model

Abbreviated title: Lack of age-related brain pathology in Octodon degus

Mathieu Bourdenx[1,2]†, Sandra Dovero[1,2]†, Marie-Laure Thiolat[1,2], Erwan Bezard[1,2] and Benjamin Dehay[1,2#]

Supplementary Information Files:

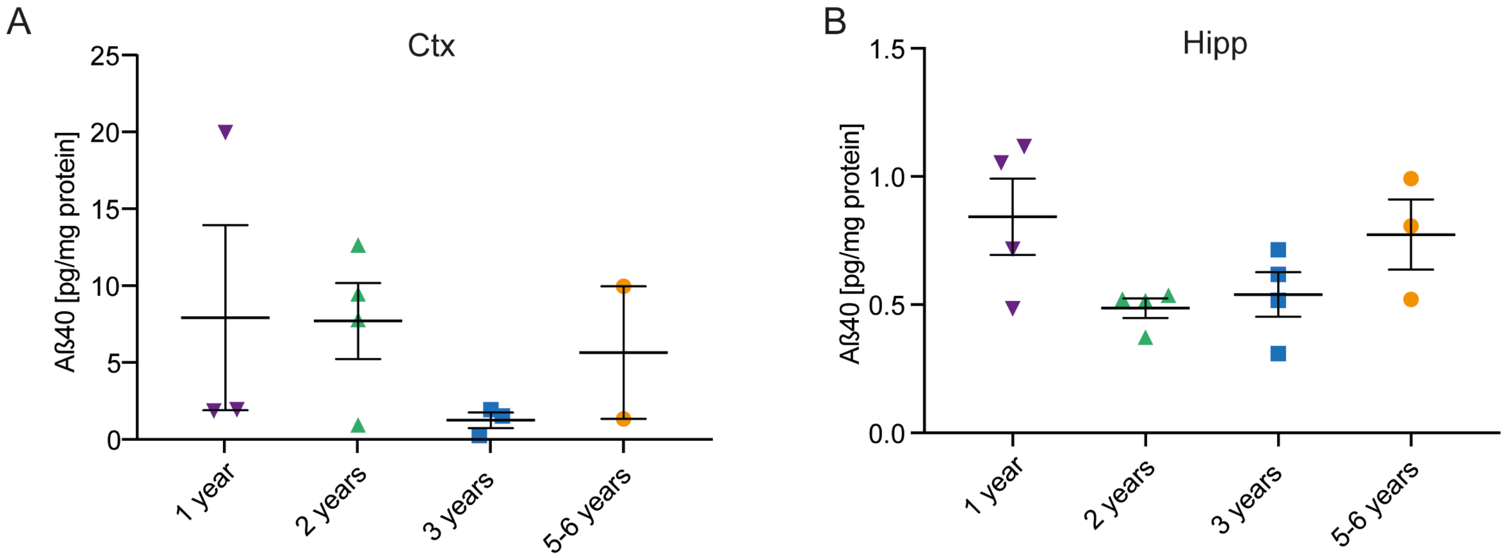
A
Ctx
Aß40 [pg/mg protein]
25
20
15
10
5
0
1 year
2 years
3 years
5-6 years
B
Hipp
Aß40 [pg/mg protein]
1.5
1.0
0.5
0.0
1 year
2 years
3 years
5-6 years

Supplementary Figure 1. Absence of Alzheimer’s like amyloid pathology in cortex and hippocampus of aged octodons. (A-B) Relative levels of Aβ40 quantified by ELISA in **(A)** cortical and **(B)** hippocampal tissue homogenates.

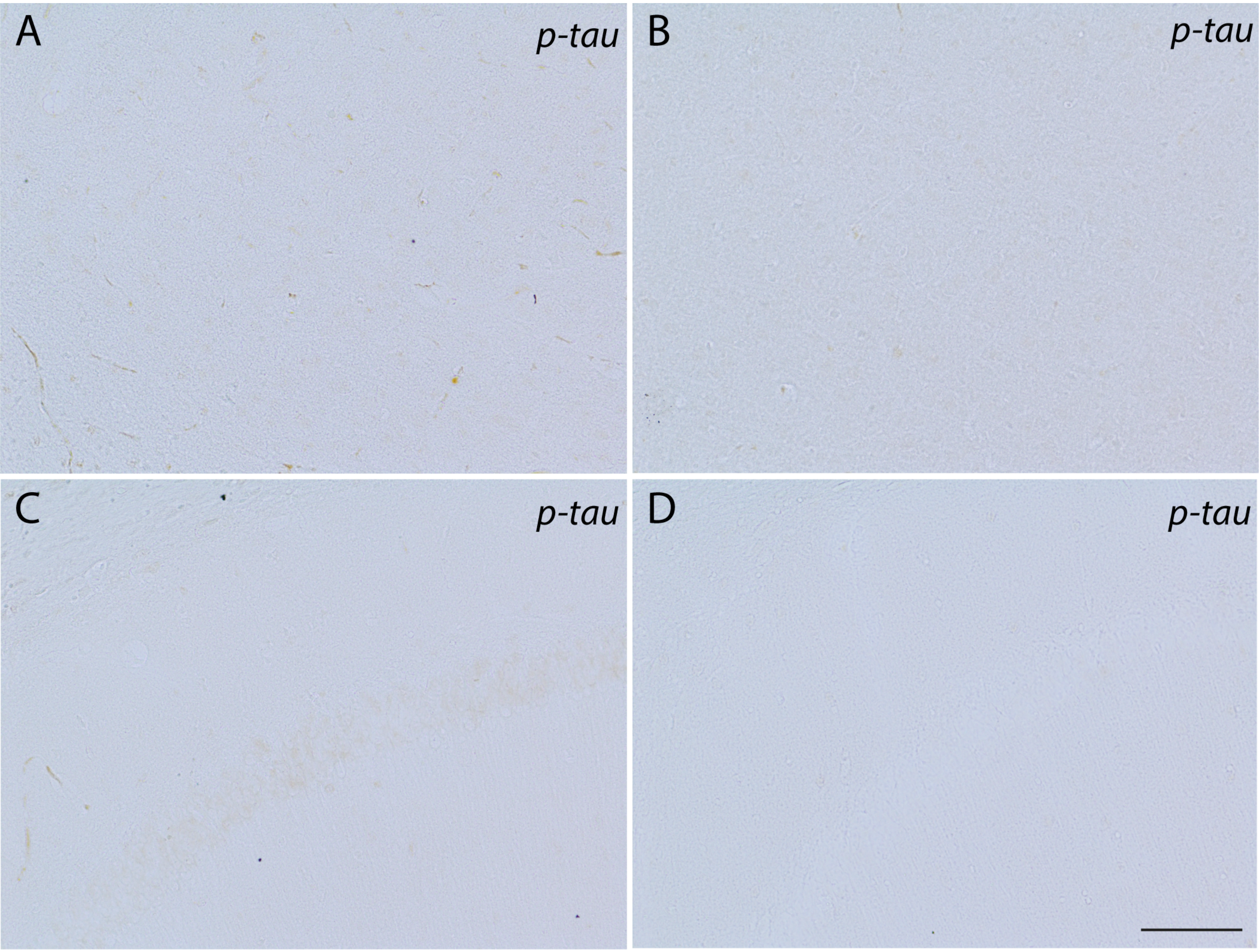
A
p-tau
B
p-tau
C
p-tau
D
p-tau

Supplementary Figure 2. Distribution of phosphorylated-tau immunostaining in the cortex and hippocampus of aged octodons. (A-D) Representative pictures of hyperphosphorylated tau (p-tau) immunostaining in the cortex of 1-year-old (**A**), 2 years-old (**B**), 3 years-old (**C**), 5-6 years-old (**D**) octodons. Scale bar: 100µm.

Species	Residues							Sequence Identity
	53	87	100	103	107	121	122	
H. sapiens	A	S	L	N	A	N	N	
M. musculus	T	N	M	G	Y	G	S	133/140 (95%)
R. norvegicus	T	N	M	G	Y	S	S	133/140 (95%)
O. degus **(predicted sequence)**	T	N	L	S	S	N	S	135/140 (96%)

Supplementary Table 1. Comparison of human and rodent α-synuclein sequences. All amino-acids differing from human sequence are shown. Blue background highlight variations compared to human sequence but identity between rodents. Orange background highlight octodon-specific variations compared to human sequence. Sequence identity is expressed compared to human sequence. Accession number are: NP_000336.1 (*H. sapiens*), NP_001035916.1 (*M. musculus*), NP_062042.1 (*R. norvegicus*), XP_004648456.1 (*O. degus*).

Variable name	Figure	PC1
TH in the striatum	1B	4.4338869
TH on actin ratio	1D	0.1282189
Syn in the Whole brain	2B	**13.8916545**
Syn in the SN	2C	3.3520295
Syn in the Striatum	2D	**11.2880522**
Syn in the OF cortex	2E	1.5116652
Syn in the motor cortex	2F	3.6626600
p-syn in the Amygdala	4E	4.8705128
p-syn in the SN	4F	**16.0645386**
p-syn in the Striatum	4G	7.6355960
p-syn in the OF cortex	4H	8.5947736
Dark p-syn in the OF cortex	4I	4.6157503
Number of Dark p-syn puncta in the OF cortex	4J	6.4038347
Poly Ub ratio in the SN	5B	4.0868168
Poly Ub ratio in the Striatum	5C	4.1165445
Poly Ub ratio in the cortex	5D	1.2217323
Abeta40 levels in the cortex	Supp. 1A	3.6580056
Abeta40 levels in the hippocampus	Supp. 1B	0.4637277

Supplementary Table 2. Variable contributions to principal component 1. The three strongest contributors are highlighted in bold. TH: tyrosine hydroxylase. Syn: α-synuclein staining. SN: substantia nigra. OF: orbito-frontal. Poly Ub: poly-ubiquitinated chains. Psyn: S129 phosphorylated α-syn. Supp.: supplementary figure
